# Population dynamics and characteristics of *Klebsiella pneumoniae* from healthy poultry in Norway

**DOI:** 10.3389/fmicb.2023.1193274

**Published:** 2023-05-18

**Authors:** Håkon Kaspersen, Anne Margrete Urdahl, Fiona Valerie Franklin-Alming, Hanna Karin Ilag, Marit A. K. Hetland, Eva Bernhoff, Iren H. Löhr, Marianne Sunde

**Affiliations:** ^1^Section for Food Safety and Animal Health, Norwegian Veterinary Institute, Ås, Norway; ^2^Section for Microbiology, Norwegian Veterinary Institute, Ås, Norway; ^3^Department of Medical Microbiology, Stavanger University Hospital, Stavanger, Norway; ^4^Department of Biological Sciences, Faculty of Mathematics and Natural Sciences, University of Bergen, Bergen, Norway; ^5^Department of Clinical Science, Faculty of Medicine, University of Bergen, Bergen, Norway

**Keywords:** *Klebsiella*, poultry, broiler, turkey, sequence types, ST4710

## Abstract

*Klebsiella pneumoniae* is an important opportunistic pathogen widely studied in relation to human infection and colonization. However, there is a lack of knowledge regarding other niches that *K. pneumoniae* may inhabit. *K. pneumoniae* isolated from healthy broiler and turkey flocks in Norway in 2018 have previously been described with regard to population structure, sequence types (STs), and the presence of virulence- and antimicrobial resistance (AMR) genes. In the present study we aimed to evaluate the dynamics of the *K. pneumoniae* population in poultry over time, with regards to AMR and virulence, and with a special focus on persistence of STs. A total of 391 flocks sampled in 2020 were included in the present study, of which 271 were from broiler flocks and 120 from turkey flocks. Similar to findings from 2018, the occurrence of *K. pneumoniae* was significantly higher based on culturing in turkey flocks (62.5%) compared to broiler flocks (24.0%). Major STs in 2020 included ST5827 (*n* = 7), ST37 (*n* = 7), ST370 (*n* = 7), ST17 (*n* = 5), and ST4710 (*n* = 5). Several STs persisted over time in both host species, including ST35, ST37, ST590, and ST17. This persistence may be due to local re-circulation or reintroduction from parent flocks. Of these five major STs, only ST590 carried AMR genes, indicating that the persistence was not associated with the presence of AMR genes. An ST4710 strain with a hypervirulence-encoding plasmid (p4710; *iro5*, *iuc5*) was recovered from turkeys in 2018. The same strain was present in turkeys in 2020, but the plasmid had lost the salmochelin locus. This loss may be attributed to reductive evolution due to the presence of several siderophores within the same isolates. In this study we also characterized a clinical ST4710 isolate from a turkey with airsacculitis. The isolate was closely related to two intestinal ST4710 isolates from healthy turkeys in 2018. These three isolates were sampled within the same location and time frame in 2018, and all carried the full p4710 virulence plasmid. These findings highlight the transmission- and infectious potential of ST4710 in turkeys.

## 1. Introduction

*Klebsiella pneumoniae* is an important opportunistic pathogen and a major public health concern. *K. pneumoniae* is known to disseminate within the hospital environment and cause opportunistic infections in vulnerable patients, but may also cause severe community-acquired infections such as liver abscesses and pneumonia. *K. pneumoniae* is separated into seven subspecies, all part of the *K. pneumoniae* species complex (KpSC), where *K. pneumoniae* subspecies *pneumoniae* has been found to have the highest public health burden (Wyres et al., 2020). Several clones of *K. pneumoniae* have been defined as global problem clones (Wyres et al., 2020). These are generally separated into two groups, hypervirulent- and multidrug-resistant clones, with little overlap between the two groups. However, convergence of virulence and multidrug-resistance has been described and is of major public health concern (Lam et al., 2019; Lan et al., 2021).

Virulence in *K. pneumoniae* is often associated with the presence of the siderophores aerobactin, salmochelin, and/or yersiniabactin, in addition to the genotoxin colibactin and the hypermucoviscosity-encoding locus *rmpADC*. Aerobactin, salmochelin, yersiniabactin, and colibactin are siderophore systems that produce iron-chelating molecules, enabling iron-scavenging from various sources (Nassif and Sansonetti, 1986; Carniel, 2001; Müller et al., 2009; Putze et al., 2009). Aerobactin has been identified as the major contributor to virulence (Matono et al., 2022), even in the presence of additional virulence factors (Russo et al., 2014, 2015). The aerobactin, salmochelin, and yersiniabactin loci are classified into several subtypes (Lam et al., 2021), making it possible to track different lineages of each locus. The aerobactin and salmochelin loci are mobilized by both conserved and diverse plasmid structures (Lam et al., 2018b), while yersiniabactin is mobilized by an integrative conjugative element often found on the chromosome (Lam et al., 2018a).

*Klebsiella pneumoniae* is well studied in relation to human infection. However, there is a lack of knowledge regarding the presence and pathogenicity of *K. pneumoniae* in other niches. Gaining insight into other niches that might harbor *K. pneumoniae* is of public health interest and helps us to understand the dynamics of this bacterium in a One Health perspective. In 2018, we isolated *K. pneumoniae* from cecal samples from healthy broiler and turkey flocks in Norway, and characterized the isolates with regard to population structure, antimicrobial resistance, and virulence genes (Franklin-Alming et al., 2021). A higher occurrence of *K. pneumoniae* was detected in the turkey flocks compared to broiler flocks, and specific sequence types (STs) were predominant among the isolates, including ST35 and ST37. Whether these STs persisted and dominated over time could not be determined in the 2018 study as sampling was performed within a single year.

In the 2018 study, a clonal expansion of a hypervirulent ST4710 strain was observed in the turkey samples. This clone carried yersiniabactin (*ybt16*) on the chromosome, in addition to an IncFII plasmid, p4710, harboring both aerobactin (*iuc5*) and salmochelin (*iro5*), contributing to a hypervirulent phenotype. *K. pneumoniae* is occasionally associated with infections in animals, including poultry. An ST4710 isolate was also detected from a clinical air sac sample submitted to our diagnostic laboratory in 2018, from a turkey in a flock with several cases of airsacculitis. Microbiological analysis revealed rich growth of mixed flora with *Escherichia coli, K. pneumoniae* and *Candida krusei*, indicating an intestinal origin of the infection.

The aim of the present study was to explore potential shifts in the population dynamics, virulence, and antimicrobial resistance of *K. pneumoniae* isolates from broiler and turkey flocks over time by comparing isolates from 2018 to 2020. Lastly, we wanted to characterize the clinical ST4710 isolate and compare it to intestinal ST4710 isolates from healthy birds.

## 2. Materials and methods

### 2.1. Sampling and laboratory analysis

In 2020, samples from broiler and turkey flocks were included in the Norwegian monitoring program for antimicrobial resistance in the veterinary sector (NORM-VET). The animals were sampled after routine slaughter under the auspices of NORM-VET. The samples were screened for *K. pneumoniae* in the present study. Overall, sampling and screening was conducted as previously described for the 2018 samples (Franklin-Alming et al., 2021). Briefly, cecal samples were taken from healthy animals at slaughter and sent to the laboratory for culturing. Ten ceca from each flock were homogenized into one sample, and were plated onto Simmons Citrate Agar with 1% Inositol (SCAI) for detection of *Klebsiella* spp. Putative *Klebsiella* spp. colonies were verified with matrix assisted laser desorption time of flight mass spectrometry (MALDI-TOF MS, Microflex Biotyper), one colony per sample, using the MBT Compass Library Revision K (2022). A total of 391 samples were screened for the presence of *Klebsiella* spp., where 271 were from broiler flocks and 120 were from turkey flocks. A subset of 115 verified KpSC isolates were subjected to Illumina sequencing as previously described (Franklin-Alming et al., 2021), 55 from broilers and 60 from turkeys. The remaining isolates were unfortunately not available for further investigation. However, the set of isolates included covered the whole sampling year. Additionally, one *K. pneumoniae* isolate from a clinical turkey air sac sample in 2018 was included.

### 2.2. Bioinformatic analysis

#### 2.2.1. Short read sequencing

All reads were quality controlled and assembled as previously described (Franklin-Alming et al., 2021). Briefly, raw reads were quality controlled with FastQC^1^ and trimmed with TrimGalore^2^ version 0.6.4. The trimmed sequences were assembled with Unicycler (Wick et al., 2017) version 0.4.8. Assemblies were quality controlled with QUAST (Gurevich et al., 2013) version 5.0.2.

Both datasets were run through Kleborate (Lam et al., 2021) version 2.2.0 to detect STs, virulence-, and antimicrobial resistance (AMR) determinants. ALPPACA (Kaspersen and Fiskebeck, 2022) version 2.0.3 was used to run a core gene phylogenetic analysis, which included all isolates from the 2018 and 2020 datasets. Briefly, all assemblies were annotated with Prokka (Seemann, 2014) version 1.14.5, followed by a pangenome analysis with Panaroo (Tonkin-Hill et al., 2020) version 1.2.9. Snp-sites (Page et al., 2016) version 2.5.1 was used to filter out constant sites from the alignment, and snp-dists version 0.8.2 was used to calculate single nucleotide polymorphism (SNP) distances. Lastly, IQTree (Minh et al., 2020) version 2.2.0.3 was used to infer the phylogeny, using ultrafast bootstrapping (Hoang et al., 2018) and GTR + F + I as the substitution model.

Genomes of ST4710 and ST37 were run on separate phylogenetic analyses, using the core genome track of ALPPACA. ST37 was selected for further analysis because this was the ST with the highest diversity, and ST4710 was selected due to the previously identified hypervirulent clone. Other STs that intermingled within the ST37 clade were also included in the phylogeny. For both phylogenies, the closest ST identified within the core gene tree was used as an outgroup. Briefly, a core genome alignment was generated with ParSNP (Treangen et al., 2014) version 1.6.1, using the largest genome among the input genomes as reference. Recombinant areas were detected with Gubbins (Croucher et al., 2015) version 3.1.6. The recombinant areas were masked from the alignment with Maskrc-svg^3^ version 0.5, followed by filtering out constant sites, snp-distance calculation, and phylogenetic inference as described above. The maximum likelihood trees were interpreted in conjunction with proprietary flock metadata, such as farm location and producer identification numbers.

Reads from all ST4710 genomes from 2018 and 2020 were mapped against the p4710 plasmid from 2018 (accession number MW316656) using BWA (Li and Durbin, 2009) version 0.7.17, SAMtools (Li et al., 2009) version 1.9, and BEDtools (Quinlan and Hall, 2010) version 2.27.1.

#### 2.2.2. Long read sequencing

One representative isolate of ST4710 from 2020, selected based on draft assembly statistics, and the clinical ST4710 isolate were subjected to long-read Nanopore sequencing. DNA was extracted from pure cultures with the GenFind V3 kit (Beckman Coulter Life Sciences) and barcoded with the SQK-LSK109 barcoding kit. The sequencing was performed using a MinION flow cell (R9.4.1) on a GridION instrument (Oxford Nanopore Technologies).

The raw Nanopore data were basecalled and demultiplexed with Guppy^4^ version 5.0.16, using the super-accurate basecalling model. The basecalled Nanopore reads were then filtered with Filtlong^5^ version 0.2.0, removing all reads shorter than 1 kbp and discarding 10% of the lowest-quality reads. Hybrid assemblies were generated using both the Nanopore and Illumina reads with Unicycler (Wick et al., 2017) version 0.5.0.

The complete plasmid sequence with both aerobactin and salmochelin loci was extracted from both hybrid assemblies and compared to the p4710 plasmid from 2018. All three plasmid sequences were annotated with Bakta (Schwengers et al., 2021) version 1.6.1, database version 4, using the “complete” parameter. Plasmid synteny between the three plasmids was compared with minimap2 (Li, 2018) version 2.22, and the comparison was visualized in R (R Core Team, 2021) version 4.2.2, using the gggenomes package (Ankenbrand, 2022) version 0.9.5.9000. Mobile Element Finder (Johansson et al., 2021) version 1.0.3, database version 1.0.2, in addition to ISFinder,^6^ was used to identify and characterize potential mobile elements within the three plasmid sequences.

### 2.3. Statistical analysis

All statistical analyses were performed with R. Differences in the occurrence of *K. pneumoniae* between hosts were calculated using χ^2^-tests. Confidence intervals (CIs) were calculated by using binomial tests with a 95% CI. Difference in within-ST and within-ST-host SNP distance was calculated with two-sided Wilcoxon-tests using Bonferroni-correction, using a 95% CI.

## 3. Results

### 3.1. Occurrence of *Klebsiella*

**FIGURE 1:**
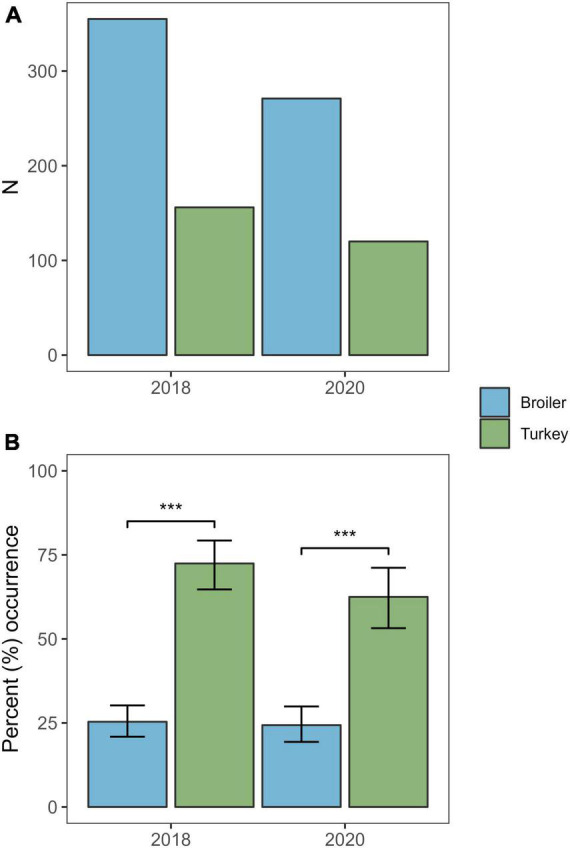
Number of samples and the occurrence of *Klebsiella pneumoniae* from broiler and turkey flocks in 2018 and 2020. **(A)** Total number of samples screened per host species per year. **(B)** Percent occurrence of *K. pneumoniae* per host species per year. Error bars represent 95% confidence intervals. ***p < 0.01.

Overall, *Klebsiella* spp. were detected in 145 of the 391 samples (37.1%) from 2020, of which 141 isolates (36.1%) were confirmed as KpSC by MALDI-TOF. Three (2.0%) of the 141 KpSC isolates were identified as *K. variicola*, all from broiler flocks. The occurrence of KpSC was 62.5% (*n* = 75/120) in turkey flocks (95% CI: 53.2–71.2) and 24.4% (*n* = 66/271) in broiler flocks (95% CI: 19.4–29.9). The occurrence was significantly higher in turkey flocks compared to broiler flocks [χ^2^(1, *N* = 390) = 21.451, *p* < 0.01] (Figure 1). A total of 115 of the 141 KpSC isolates were subjected to Illumina sequencing. Full Kleborate results for all isolates analysed in this study can be found in Supplementary Table 1.

### 3.2. AMR- and virulence genes

AMR genes were detected in 13 of the 115 KpSC isolates (11.3%); in six (9.8%) turkey and seven (13.0%) broiler isolates. Resistance genes for tetracycline occurred in seven isolates (6.1%), specifically *tetA* (*n* = 4), *tetD* (*n* = 2), and *tetB* (*n* = 1), followed by resistance to fosfomycin in four isolates (3.5%), all *fosA7*, and beta-lactams in three isolates (2.6%), all *bla*_*TEM–*1–D_.

Overall, virulence genes were identified in 15 isolates (13.0%), where 13 were from turkeys and two from broilers. Yersiniabactin was identified in 15 isolates (13.0%). Five different lineages of yersiniabactin were detected: *ybt5* (*n* = 2), *ybt10* (*n* = 1), *ybt14* (*n* = 3), *ybt15* (*n* = 3), and *ybt16* (*n* = 6). Aerobactin ST (AbST) 94, lineage *iuc5*, was detected in five (4.3%) isolates. These all belonged to ST4710, and in addition carried yersiniabactin *ybt16*, yersiniabactin ST (YbST) 391, mobilized on an ICE*Kp*12. Colibactin and *rmpA* were not detected in any of the isolates.

### 3.3. Sequence types and core gene phylogeny

A total of 62 STs were detected among the 115 isolates (Simpson diversity 0.982), where 39 (62.9%) were only represented by a single isolate. The major STs in 2020 included ST5827 (*n* = 7), ST37 (*n* = 7), ST370 (*n* = 7), ST17 (*n* = 5), and ST4710 (*n* = 5). Six of the seven isolates of ST5827 were from turkey flocks. ST370 and ST4710 were only detected in turkey flocks, while ST37 and ST17 were detected in both broiler and turkey flocks. Several STs detected in 2020 were also present in 2018 (*n* = 27), including ST37, ST4710, ST35, ST17, and ST590. Table 1 presents the AMR and virulence genes present in the major STs detected in both years.

**TABLE 1:** Comparison of virulence- and antimicrobial resistance genes present in each major *Klebsiella pneumoniae* sequence type from turkey and broiler flocks in 2018 and 2020.

				Virulence loci						AMR genes	
**ST**	**Year**	**Host**	**N**	***ybt10***	***ybt8***	***ybt15***	***ybt16***	***iro5***	***iuc5***	***fosA7***	***tetB***
ST17	2018	Broiler	2	1	0	0	0	0	0	0	0
ST17	2020	Broiler	3	0	0	0	0	0	0	0	0
ST17	2018	Turkey	3	2	0	0	0	0	0	0	0
ST17	2020	Turkey	2	0	0	0	0	0	0	0	0
ST35	2018	Broiler	4	0	2	0	0	0	0	0	0
ST35	2018	Turkey	24	0	0	0	0	0	0	0	1
ST35	2020	Turkey	1	0	0	0	0	0	0	0	0
ST37	2018	Broiler	5	0	0	0	0	0	0	0	0
ST37	2020	Broiler	4	0	0	0	0	0	0	0	0
ST37	2018	Turkey	6	0	0	6	0	0	0	0	0
ST37	2020	Turkey	3	0	0	3	0	0	0	0	0
ST590	2018	Broiler	2	0	0	0	0	0	0	2	0
ST590	2020	Broiler	2	0	0	0	0	0	0	2	0
ST590	2018	Turkey	7	0	0	0	0	0	0	7	0
ST590	2020	Turkey	1	0	0	0	0	0	0	1	0
ST4710	2018	Turkey	16	0	0	0	14	16	16	0	0
ST4710	2020	Turkey	5	0	0	0	5	0	5	0	0

The column N denotes the number of isolates. The columns under virulence and AMR denote the number of isolates harboring the respective loci/genes. ST, sequence type; AMR, antimicrobial resistance.

**FIGURE 2:**
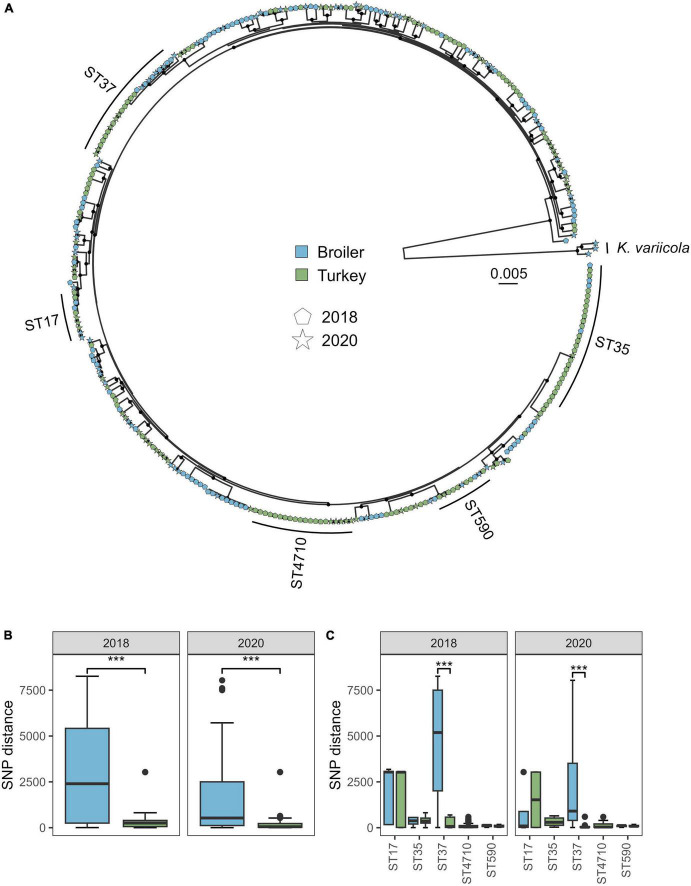
Phylogeny and SNP distance statistics of *Klebsiella pneumoniae* from broiler and turkey flocks in 2018 and 2020. **(A)** Core gene maximum likelihood phylogeny of 318 *K. pneumoniae* isolates from broiler and turkey flocks in 2018 and 2020. Tip colors represent host species, and tip shapes represent sampling year. The major STs are marked. Black dots on nodes represent accepted bootstrap values (=0.95). The tree is midpoint rooted. **(B)** Within-ST SNP distances per year for isolates within the same host species, aggregated for the five major STs. ***p < 0.01. **(C)** Within-ST SNP distances per year for each major ST and host species.

The pangenome analysis identified 15,211 genes among the 318 isolates from both 2018 and 2020, of which 4126 were core genes. The core gene alignment used in the phylogenetic analysis was 4.14 Mbp long, where 90.14% were constant sites. A total of 408,462 variable sites were detected in the alignment. The phylogenetic tree revealed several deep-branching lineages within the *K. pneumoniae* subsp. *pneumoniae* clade (Figure 2A). The within-ST distance was higher in broilers compared to turkeys in both years, when looking at the distances for the five major STs marked in the tree (ST17, ST35, ST37, ST590, and ST4710, Figure 2B) (*W* = 96030, *p* < 0.01). The within-ST SNP distance for ST37 was significantly lower in turkeys compared to broilers, for both 2018 (*W* = 580, *p* < 0.01) and 2020 (*W* = 72, *p* < 0.01) (Figure 2C). ST1779 (*n* = 6) and ST4718 (*n* = 1), both single locus variants of ST37, intermingled on the same clade as the ST37 isolates, and were therefore included in the core genome phylogeny below.

### 3.4. ST37 core genome phylogeny

**FIGURE 3:**
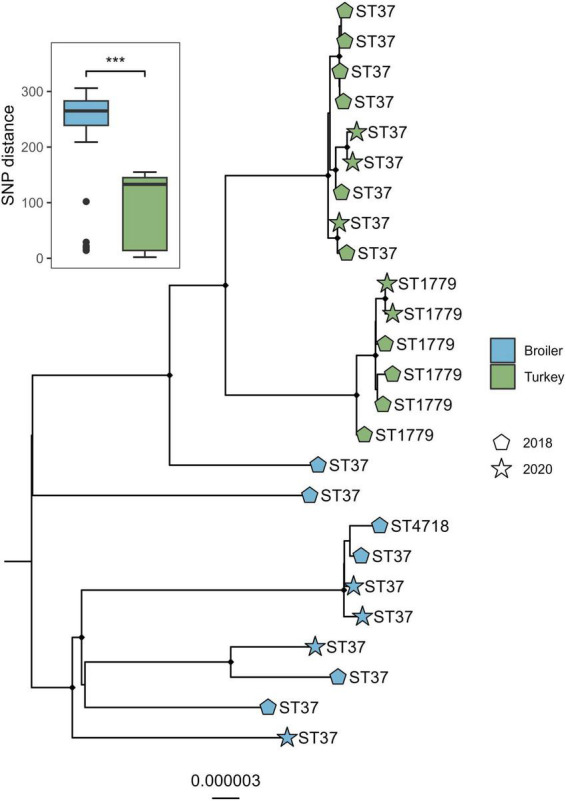
Core genome maximum likelihood phylogeny of 18 *Klebsiella pneumoniae* ST37 and closely related single locus variants from broiler and turkey flocks in 2018 and 2020. Boxplot represent the within-host SNP distances. Tip labels represent the sequence types, shapes represent year of sampling, and tip point color represent host species. Black dots on nodes represent accepted bootstrap values (=0.95). The tree is outgroup-rooted on the closest sequence type. ***p < 0.01.

The alignment of the genomes from the ST37 clade and one outgroup of ST394 was 4.88 Mbp in size, which accounted for on average 91.1% of the total sizes for each genome included. A total of 438 variable sites were detected. The isolates clustered according to host species and ST (Figure 3), where the SNP distances detected within broiler isolates were significantly higher compared to distances detected within isolates from turkeys (*W* = 17128, *p* < 0.01). Both sampling years intermingled within each host species clade in the tree.

### 3.5. ST4710 clone and virulence plasmid

**FIGURE 4:**
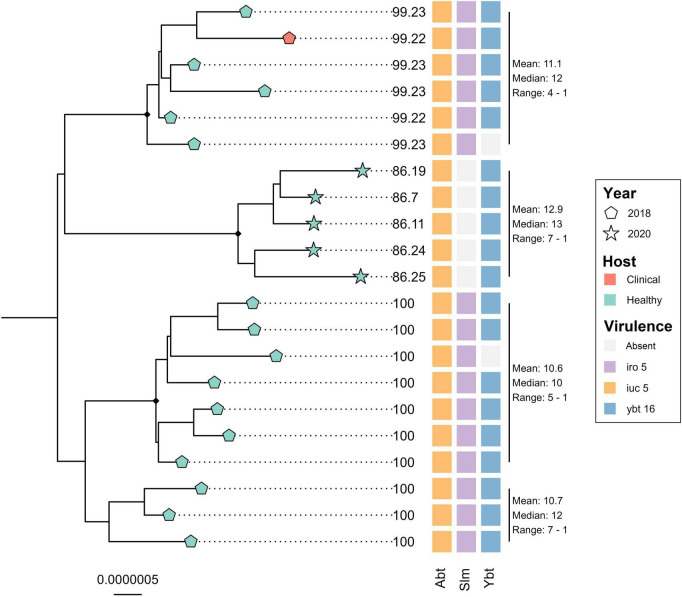
Core genome maximum likelihood phylogeny of 21 *Klebsiella pneumoniae* ST4710 from turkey flocks in 2018 and 2020. Tip point shapes represent year of sampling, and color represent host status. Tip labels represent the percent coverage of the reference plasmid p4710 (MW316656) for each isolate. SNP distance is summarized for each major clade. Black dots on nodes represent accepted bootstrap values (=0.95). The tree is outgroup- rooted on the closest sequence type. Abt, aerobactin; Slm, salmochelin; Ybt, yersiniabactin.

The core genome alignment of the 21 ST4710 genomes and one outgroup genome belonging to ST290, was 4.77 Mbp long, which accounted for an average of 87.1% of the total sizes for each genome included. A total of 102 variable sites were detected in the alignment. Figure 4 shows the core genome maximum likelihood phylogeny of the ST4710 isolates rooted on the outgroup genome, in addition to occurrence of virulence genes. All isolates clustered according to year of sampling. The clinical turkey isolate clustered together with two isolates from healthy turkeys, with a median SNP distance of 12. These three isolates originated from the same farm, and were sampled within the same month. Five *K. pneumoniae* isolates from the 2020 turkey flocks were classified as ST4710, and all five carried the aerobactin and yersiniabactin loci, but not the salmochelin loci. The clinical isolate from 2018 carried all three. Hybrid assemblies revealed the presence of an IncFII plasmid in both the clinical and the 2020 isolate, with a size of 206.7 Kbp and 177.99 Kbp, respectively. Read mapping to the p4710 plasmid from 2018 showed an average sequence coverage of 86.3% for the five 2020 isolates, and 99.2% for the clinical isolate.

**FIGURE 5:**
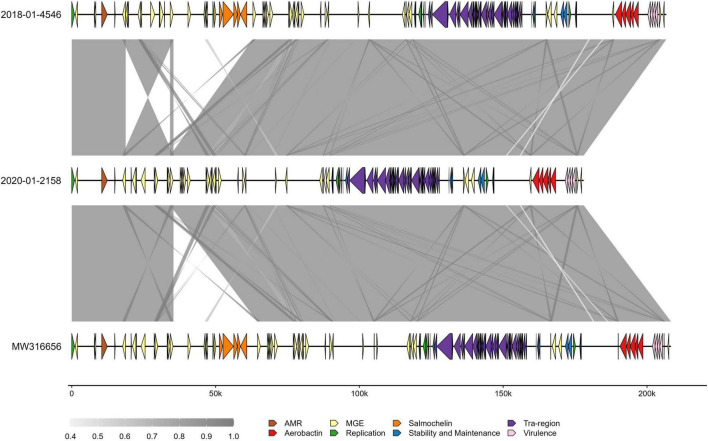
Comparison of three ST4710 virulence plasmids in *Klebsiella pneumoniae* isolated from turkey flocks in 2018 and 2020. Gray-shaded areas represent percent identity between sequences. Colors on arrow segments represent different gene types, as specified in the inset legend. Genes that did not fall into the categories presented have been removed for ease of interpretation. MGE, mobile genetic elements; 2018-01-4546, plasmid from clinical isolate; 2020-01-2158, plasmid from *K. pneumoniae* isolated from healthy turkey sampled in 2020; MW316656, reference plasmid from the 2018 study.

Comparing the clinical isolate and the 2020 isolate to the p4710 plasmid from 2018 revealed a ∼30 kb deletion of an area harboring the salmochelin operon in the 2020 isolate (Figure 5). This area was part of ISKpn26, an IS5-family composite transposon, with 100% sequence coverage of flanking mobile genetic elements.

## 4. Discussion

This study aimed to evaluate *K. pneumoniae* ST dynamics and presence of AMR- and virulence determinants in broiler and turkey flocks over time by comparing genomes from 2018 to 2020. Overall, we identified a high diversity of *K. pneumoniae* among broiler- and turkey flocks in Norway both in 2018 (Franklin-Alming et al., 2021) and 2020. Several STs were detected both in 2018 and 2020, some of which were more diverse in broilers than in turkeys. Further, we discovered that the ST4710 hypervirulent clone observed in turkeys in 2018 was also present, but less prevalent in turkeys in 2020. However, the ST4710 isolates from 2020 lacked the salmochelin operon. Lastly, we characterized a clinical air sac ST4710 isolate, and found that it was closely related (<12 SNPs) to the intestinal ST4710 isolates from 2018.

The major STs previously identified in 2018 were still dominating in 2020. This indicates persistence of these major STs within the poultry production. This may be due to local re-circulation or introduction through the poultry production chain from parent flocks. The overall within-ST diversity for these major STs was higher in broilers compared to turkeys. This was also shown for the ST37 clade. These findings were supported by the result from the core genome analysis, which included a much bigger portion of each genome, and the inclusion of the locus variant STs within the same clade. The clustering by host species identified in ST37 suggest little to no overlap of this ST between these two animal niches. For ST37, the smallest within-host SNP distances were detected in the turkey samples, where both sampling years intermingled within the same clade. This suggests that ST37 is persisting in the turkey population within the study period. The high similarity between the turkey ST37 isolates can likely be attributed to clonal expansion. This expansion may be due to reintroduction from higher up in the poultry production pyramid, which has been previously described for antimicrobial resistant *E. coli* in the Norwegian poultry production (Mo et al., 2014; Kaspersen et al., 2020a,b).

The lower diversity seen in ST37 from turkeys may also be explained by the population and production differences between turkeys and broilers. The turkey production in Norway is small with only about 50 farms all together producing about 950,000 turkeys, while the broiler production is much larger with about 500 farms producing more than 74 million broilers (Falk et al., 2022). The sampling strategy used in NORM-VET was the same for the 2 years, therefore the results from the 2 years should be comparable. However, the population and production differences may have an impact on the detected diversity between the two species in the study. With a sampling design aiming at sampling 300 random broiler flocks per year, there will be very few broiler farms from which samples are taken from more than one flock. As a contrast to samples from turkey, where almost all produced turkey flocks are sampled per year, and therefore consist of samples from the same farm though from several flocks. In addition, due to the relatively small turkey population, several of the turkey flocks probably share the same parent animal flock, while this is less of a concern in broilers. There is also a difference with regard to time of slaughter and thereby age of sampling between the two populations. Broilers are slaughtered at approximately 30 days of age, while turkeys are slaughtered at 87–130 days (Animalia, 2019). All these aspects may have had an impact on the results, and thereby explain the difference in occurrence of *K. pneumoniae* between the two species.

Several STs from Norwegian poultry overlap with STs isolated from both healthy and sick humans in Norway, such as ST35, ST37, and ST17 (Raffelsberger et al., 2021; Fostervold et al., 2022; Hetland et al., 2023a). AMR- and virulence genes were scarce within each of these STs in the current study. However, some of the ST35 isolates from both human clinical samples and carriage studies harbored yersiniabactin *ybt8*, similar to the two yersiniabactin-positive ST35-isolates from the current study, indicating that there might be a potential for overlap between the human and poultry niches. This is currently being followed up in an ongoing study investigating the cross-talk of *K. pneumoniae* between several niches and host species, such as humans, animals, and the environment (Hetland et al., 2023b).

*K. pneumoniae* subsp. *pneumoniae* was identified as the main subspecies within the culture positive samples from broilers and turkeys, in both 2018 (Franklin-Alming et al., 2021) and 2020. Only three samples from broilers contained *K. variicola* subsp. *variicola*. This indicates that *K. pneumoniae* subsp. *pneumoniae* is the overall dominant KpSC subspecies in the gut of poultry. Studies on *K. pneumoniae* from healthy poultry are scarce, making it difficult to compare results, but our findings are in line with studies on carriage in healthy humans in Norway, where *K. pneumoniae* subsp. *pneumoniae* was also the dominant subspecies (Raffelsberger et al., 2021). Additionally, *K. pneumoniae* subsp. *pneumoniae* is the dominant subspecies reported in human blood stream- and urinary tract infections (Fostervold et al., 2022) and within the gut of healthy pigs in Norway (Kaspersen et al., 2023). A previous study hypothesized that the molecular determinants of infection are similar between humans and animals (Brisse and van Duijkeren, 2005). The observations within the current study and the above-mentioned studies seem to concur with this hypothesis. Further work with a One Health perspective and focus on cross-sectional sampling is needed to further investigate this hypothesis.

Infections with *K. pneumoniae* are sporadically identified in animals (Brisse and van Duijkeren, 2005; Bidewell et al., 2018). There is, however, limited knowledge on the pathogenic potential of *K. pneumoniae* in poultry. In the present study, the hypervirulent clone ST4710 persisted within the turkey production during the study period. Moreover, the clinical ST4710 isolate from 2018 was closely related to the other ST4710 from healthy animals in 2018, and to some extent in 2020. Healthy carriers of ST4710 were also identified from the same farm within the same time frame as the clinical ST4710 isolate was detected. This indicates that the ST4710 clone identified within the cecum of healthy turkeys has the potential to cause infections. The pathogenicity of ST4710 may be due to the presence of the p4710 plasmid, which was present within all the ST4710 isolates. Interestingly, the salmochelin locus was lost in the 2020 isolates, and may be a result of evolutionary adaptation of *K. pneumoniae* within the turkey gut. Of the most common siderophores in hypervirulent *K. pneumoniae*, aerobactin is regarded as a critical virulence factor for hypervirulent *K. pneumoniae* (Russo et al., 2015). In contrast, salmochelin was found to be the least important siderophore. The loss of the salmochelin locus in the 2020 ST4710 isolates may therefore be related to fitness, since salmochelin does not contribute significantly to siderophore production in the presence of the aerobactin locus (Russo et al., 2015). The presence of a yersiniabactin locus in the ST4710 isolates from both 2018 and 2020 may also support this hypothesis, as the siderophore yersiniabactin has also been found to have a higher activity compared to salmochelin (Russo et al., 2015). According to the theory of bacterial streamlining, bacteria evolve by reductive evolution, and thus try to reduce their genome size for efficient nutrient use (Giovannoni et al., 2014). The presence of both the aerobactin and yersiniabactin loci may therefore have rendered the siderophore production of salmochelin redundant and of little use to the bacterium. The loss of virulence genes in favor of the aerobactin locus has previously been described in carbapenem-resistant hypervirulent *K. pneumoniae* (Shankar et al., 2021), where the loss of *rmpA2* was detected. The presence of both *rmpA2* and the aerobactin locus within the same isolate led to slower growth (Shankar et al., 2021), increasing the fitness cost. Further studies are needed to confirm if a similar hypothesis can be applied to the salmochelin and aerobactin loci.

A comparable occurrence of AMR genes were identified in 2020 and 2018 (Franklin-Alming et al., 2021). As before, genes conferring resistance to tetracycline were the most prevalent within the poultry production, however, the overall prevalence of AMR was low in both the turkey and broiler flocks. The five major STs that persisted from 2018 to 2020 did not carry any AMR genes, except ST590, which had a similar profile as that detected in ST590 isolates in 2018. Thus, the presence of AMR genes may not be an explanatory factor for the persistence of these STs within the poultry production.

In conclusion, several STs persisted within the broiler and turkey production within the study period, the major ones being ST17, ST35, ST37, ST590, and ST4710. This persistence may be due to local re-circulation or reintroduction from parent flocks, as described in several other studies on *E. coli*. Most of the persisting STs did not carry AMR genes, and the persistence of these STs within the poultry production is therefore likely not associated with the presence of such genes. The detection of a clinical ST4710 isolate in 2018, highly similar to other ST4710 isolates identified in the gut of healthy turkeys within the same time frame and location, highlight the transmission- and infectious potential of ST4710 in turkeys. The loss of the salmochelin operon in the ST4710 isolates from 2020 may be a result of reductive evolution due to the presence of other siderophores within the same isolates.

## Data availability statement

The datasets presented in this study can be found in online repositories. The names of the repository/repositories and accession number(s) can be found in the article/Supplementary material.

## Ethics statement

Ethical review and approval was not required because the samples were taken after routine slaughter, under the auspices of the NORM-VET surveillance program.

## Author contributions

IL, AU, and MS provided the funding and performed the project administration. MS, AU, and HK conceptualized the study and wrote the initial manuscript. FF-A, HI, EB, and HK did the laboratory analyses. HK and MH analyzed the data. All authors contributed to editing the manuscript.

## Footnotes

1
https://www.bioinformatics.babraham.ac.uk/projects/fastqc/

2
https://www.bioinformatics.babraham.ac.uk/projects/trim_galore/

3
https://github.com/kwongj/maskrc-svg

4
https://nanoporetech.com/

5
https://github.com/rrwick/Filtlong

6
https://www-is.biotoul.fr/index.php
